# Labyrinthine Fluid Signal Intensity on T2-Weighted MR Imaging in Patients With Vestibular Schwannomas Undergoing Proton Radiotherapy: A Longitudinal Assessment

**DOI:** 10.1097/MAO.0000000000003774

**Published:** 2022-12-21

**Authors:** Kimberley S. Koetsier, William A. Mehan, Karen Buch, D. Bradley Welling, Peter Paul G. van Benthem, Erik F. Hensen, Helen A. Shih

**Affiliations:** ∗ Department of Otorhinolaryngology and Head and Neck Surgery, Leiden University Medical Center, Leiden, the Netherlands; † Department of Radiation Oncology, Massachusetts General Hospital; ‡ Department of Radiology, Massachusetts General Hospital; § Harvard Medical School; ∥ Department of Otolaryngology Head and Neck Surgery, Massachusetts Eye and Ear Infirmary, Massachusetts General Hospital, Boston, Massachusetts, USA

**Keywords:** 3D-CISS, Hearing disorders, Labyrinth signal loss, Proton therapy, Radiotherapy, Vestibular schwannoma

## Abstract

In vestibular schwannoma patients, a loss of signal intensity (SI) on T2-weighted magnetic resonance imaging (MRI) has been reported within the ipsilateral labyrinth. The purpose of this study was to quantitatively evaluate the occurrence and course of this intensity loss in relation to proton radiotherapy and its possible association with hearing loss.

**Study Design:**

Retrospective chart review.

**Setting:**

Tertiary referral center.

**Patients:**

Patients who received proton therapy for a vestibular schwannoma and underwent at least two high-resolution T2-weighted cisternographic sequence (constructive interference in steady state/fast imaging employing steady-state acquisition/DRIVE) MRIs and audiometry assessments.

**Main Outcome Measures:**

Relative T2 SIs from the vestibules and basal/apical cochlear turns of the labyrinth, bilaterally.

**Results:**

Ninety-five MRI scans from 34 patients were included. The apical turn of the ipsilateral cochlea showed a lower mean cochlear SI than on the contralateral side (±3.5 versus 5.0). The mean relative cochlear SI did not significantly change after proton radiotherapy. The ipsilateral vestibule showed a higher SI than the cochlea. The relative mean cochlear SI was not directly correlated to (the degree of) hearing loss before or after proton radiotherapy, nor did it predict future hearing loss.

**Conclusion:**

The relative mean cochlear SI on cisternographic T2-MRI in vestibular schwannoma patients is diminished on the treated side, when compared with the ipsilateral vestibule and the contralateral cochlea/vestibule. The SI of the ipsilateral cochlea does not further decrease after proton radiotherapy and seems to be related to the tumor rather than the therapy. The diminished cochlear SI does not correlate with subsequent loss of hearing.

## INTRODUCTION

High-resolution cisternographic T2-weighted magnetic resonance (MR) sequences, also known as balanced steady-state free precession sequences (three-dimensional constructive interference in steady state [3D-CISS], drive equilibrium radio frequency reset pulse (DRIVE), or fast imaging employing steady-state acquisition [FIESTA]) are used for imaging of the cerebellopontine angle, the internal acoustic canal, and the labyrinth (1). These imaging sequences are frequently used for the diagnosis and surveillance of vestibular schwannomas (VSs): benign tumors arising from Schwann cells on the vestibulocochlear nerve that cause hearing loss, tinnitus, and balance disorders among other symptoms. Using T2-weighted MR imaging (T2-MRI), a VS can be seen as a hypointense mass within the hyperintense (fluid-filled) cerebellopontine angle and/or the internal auditory canal. It has been observed that in VS patients, the cochlea and/or vestibule can show a decreased (intermediate) signal intensity (SI) on T2-weighted MRI, which is not directly caused by the tumor mass itself. The occurrence of this SI loss has been observed in several cross-sectional studies; however, its cause and clinical relevance are still subject of debate (2–9).

Not all VS patients show a decreased labyrinthine SI in the ipsilateral labyrinth. It is therefore likely that, at some point, the labyrinthine SIs were equal on both sides in all VS patients. The time point at which the labyrinthine signal alteration first occurs remains unknown. The course of the SI over time and the impact of *proton* radiotherapy on this intensity has not yet been quantitatively studied. Conventional photon radiotherapy contains x-rays that have their highest radiation energy deposit shortly after tissue entry and continue to irradiate the tissue beyond the target. Proton radiotherapy, on the other hand, delivers a beam of proton particles that stop at the tumor, which is a result of the low-radiation-dose entry and finite Bragg peak. Proton radiation causes—in addition to a different dose distribution—a different biological effect on the tumor and the organs at risk around it. This in theory could lead to a different labyrinthine reaction to radiation and consequently provide different SI than conventional photon irradiation. The purpose of this study is to evaluate the longitudinal changes in and between cochlear and vestibular SI on cisternographic T2-weighted MRI of VS patients undergoing proton radiotherapy, to better understand the timing of its occurrence and the impact of proton radiotherapy. The secondary aim is to evaluate the relation of the SI loss and the patients' hearing status.

Previously reported outcome measures consisted of categorized physician-rated MR SI assessments, comparing the ipsilateral labyrinth with the contralateral labyrinth or the cerebrospinal fluid SI (2–6). Although these showed good interrater reliability, the use of a (binary) classification causes loss of individual nuance, statistical power, and diminishes insight in effect size (10). To increase the sensitivity of the outcome parameters, this study will report labyrinthine SI values relative to the nonirradiated cerebellum and not only to the contralateral labyrinth. The measurement of relative MRI SI values enables comparison of different MR acquisitions and thus allows for longitudinal evaluations of both the ipsilateral and contralateral labyrinth.

## MATERIALS AND METHODS

### Patient Selection and Proton Radiotherapy

The records of patients treated for a sporadic VS with proton radiotherapy at the (Massachusetts General Hospital Francis H. Burr Proton Therapy Center (Boston, USA) were retrospectively reviewed. Patients who had undergone at least two MRIs (at least one before and after radiotherapy) and audiometry performed within 7 months of the MRI dates were eligible for this study. Radiological evaluation comprised high-resolution cisternographic T2-weighted MR sequences, such as 3D constructive interference in steady state, DRIVE, or FIESTA. Multicenter, multivendor MRI was included in the study. Patients with a previous tumor resection or neurofibromatosis type 2 were excluded.

### MRI Delineation of the Cochlea and Vestibule

For this study, T2-weighted MRI signal was normalized to the cerebellar white matter by using SI values relative to the cerebellum and by assessing different regions within the labyrinths of patients with sporadic VSs (11). The contralateral labyrinth served as an internal control group. The use of relative SI values offers the opportunity to establish a surrogate measurement error of the labyrinthine SI: by calculating the differences in the contralateral SI values within and between MRI scans. A difference between the ipsilateral and contralateral labyrinth should at least exceed this assessment margin.

Region of interest (ROI) delineation and MRI assessments were performed by using MIM software (MIM Software Inc., Beachwood, OH, USA). ROIs were delineated by experienced neuroradiologists (W.A.M. and K.B.). Assessors were blinded to the clinical data. The ROIs were placed bilaterally on the apical and basal turns of the cochlea, and on the center of the vestibular system (utricle/saccule). The assessors were instructed to select a hyperintense region within the organs. In addition, the contralateral cerebellum was selected as a reference to obtain normalized signal intensities. The cerebellum was chosen because of its localization outside of the irradiated region and because it is commonly used as a reference (11,12). The ROI was placed on the greatest dimension in the axial plane. As the cochlear SI ipsilateral to the tumor was less homogeneous than that of the contralateral cochlea, a second and larger part of the ipsilateral basal cochlea was delineated because in doing so, the measured Sis were less dependent on the specific place of delineation within the cochlea.

### Outcome Parameter Definitions

The mean and standard deviations (SDs) of the SI scores of the different ROIs were exported from MIM. Subsequently, the scores were normalized to the cerebellum to establish quantitative and more comparable outcomes between scans and patients. The relative SI of the different ROIs was calculated by dividing the mean value of the ROI by the mean/SD value of the reference ROI (cerebellum):


$$\text{Relative cochlear signal intensity}=\frac{\text{Mean cochlear signal intensity}}{\text{Mean contralateral cerebellar signal intensity}}$$

For the hearing assessment, the pure-tone average (PTA) of air conduction and maximum speech discrimination scores (SDSs) were used. Per American Academy of Otolaryngology–Head and Neck Surgery definition, PTA is defined as the average dB hearing loss at 0.5, 1, 2, and 3 kHz (average of 2 and 4 kHz, if 3 kHz was absent). Progressive SDS loss was defined as a decrease in SDS below the 95% critical difference in reference to the pretreatment SDS, to account for the mathematical uncertainties of this score (13). Because PTA has a higher sensitivity to change in hearing levels, this outcome measure was chosen over SDS for the statistical analyses.

### Statistical Analysis

First, the homogeneity of the relative mean SI from different regions within one cochlea and the vestibule was statistically compared: an intraclass correlation coefficient (ICC) was calculated to assess the overall agreement between the regions, and a paired *t* test or Wilcox rank sum test was performed to assess group-level differences. Second, the change in SI scores per patient over time was assessed. For the contralateral cochlea/vestibule, this can be seen as a surrogate for a measurement error. Third, the differences between the ipsilateral and contralateral cochleae/vestibules were assessed.

To test the association between relative cochlear SI scores and the PTA, a linear mixed model was fitted. The model was corrected for the duration of follow-up. In a second model, which includes both bilateral measurements, for the side of measurement. The two models contained a random intercept per patient and a random effect for the interaction of side and individual, respectively. A residual plot was used to assess model fit. The predictive value of the pretreatment relative SI scores for these symptoms was assessed with a Spearman's correlation between baseline cochlear SI and posttreatment hearing.

Statistical analyses were performed using RStudio Inc. v.1.2.1335 (Boston, MA) and lme4 (14). The study protocol was approved by the institutional review board.

## RESULTS

### Patient, Tumor, and Treatment Characteristics

In total, 34 patients met the inclusion criteria. Characteristics are shown in Table 1, and details on the MRI scans and mean absolute outcomes can be found in the supplemental material, http://links.lww.com/MAO/B545. Proton radiotherapy consisted of stereotactic proton radiosurgery in 11 patients and fractionated stereotactic proton radiotherapy in 23 patients. These patients were part of a cohort that has been described before (15). We evaluated MRI and audiometry before treatment and during follow-up, specifically the first available MRI and audiometry after treatment and the most recent available MRI and audiometry after treatment. The median time interval between radiotherapy and first posttreatment MRI was 14 months (interquartile range [IQR], 9–17 months); the median time interval between radiotherapy and most recent MRI was 49 months (IQR, 27–62 months). Tumor control, defined as residual disease not requiring additional (salvage) treatment, was 100% at last follow-up in this patient group.

**TABLE 1:** Patient characteristics

Age at treatment, median (range), yr	64 (47–76)
% Male	44
Median time between diagnosis and proton therapy (range), yr	1 (0–8)
Indication for treatment	26 growth 5 patient preference 3 large size
Laterality tumor, % left	53
Tumor volume, median (range), cc	0.8 (0.4–1.2)
Ipsilateral PTA, median (range), dB	41 (18, 3–75)*^a^*
Contralateral PTA, median (range), dB	18 (10, 0–39)*^a^*
Max SDS ipsilateral, median (range), %	70 (62–89)
Max SDS contralateral, median (range), %	98 (96–100)

PTA indicates pure-tone average, SDS = speech discrimination score.

*^a^*One patient only had SDS available for the baseline assessment.

### Apical Versus Basal Cochlear SI

There were two ROIs delineated within the bilateral cochleae: an apical and a basal region. Table 2 shows the mean SI values for each time point.

**TABLE 2:** Mean relative signal intensity values of the bilateral labyrinth on T2-wegithed magnetic resonance imaging sequences and the percentages of ipsilateral regions that showed a decreased signal intensity compared with the contralateral side, separated by follow-up duration

	Pretreatment			Short-Term Follow-up			Long-Term Follow-up^a^		
	Signal Intensity Score, Mean (SD)	% Decreased Signal Intensity*^b^*	Median % Decrease	Signal Intensity Score, Mean (SD)	% Decreased Signal Intensity*^b^*	Median % Decrease	Signal Intensity Score, Mean (SD)	% Decreased Signal Intensity*^b^*	Median % Decrease
Ipsilateral									
Cochlea apex ROI	3.5 (1.3)	100	22	3.5 (1.2)	100	31	3.7 (1.3)	96	30
Cochlea basal ROI	4.1 (1.7)	97	34	4.2 (1.5)	85	34	4.3 (1.9)	85	38
Vestibular ROI	5.0 (2.3); median, 4.5	79	16	5.4 (2.0); median, 4.9	69	15	5.3 (2.3); median, 5.2	74	11
Cochlea larger basal ROI^c^	3.2 (1.3)	100	61	3.3 (1.3)	97	67	3.6 (1.3)	96	61
Contralateral									
Cochlea apex ROI	5.0 (2.0)	—	—	5.2 (1.7)	—	—	5.2 (1.9)	—	—
Cochlea basal ROI	5.3 (2.1)	—	—	5.5 (2.2)	—	—	5.6 (2.0)	—	—
Vestibular ROI	5.6 (2.5); median, 5.1	—	—	6.0 (2.2); median, 5.8	—	—	6.0 (2.3); median, 5.5	—	—

Differences over time were not significant.

*^a^*Twenty-seven of 34 patients were available for the third assessment.

*^b^*Percentage of patients who demonstrated a decreased signal intensity when compared with contralateral cochlea or vestibule.

*^c^*As the cochlear signal intensity ipsilateral to the tumor was less homogeneous (Ipsilateral Cochlea section) than that of the contralateral cochlea, a second and larger part of the ipsilateral basal cochlea was delineated because in doing so, the measured signal intensities were less dependent on the specific place of delineation within the cochlea.

ROI indicates region of interest; SD, standard deviation.

#### Contralateral Cochlea

The overall mean difference between the apical and basal SI was 0.3 (SD, 1.1) and was not significant (*p* = 0.21, Wilcox-rank sum), indicating that the SI within the apical versus basal turns of the contralateral cochlea was relatively homogenous. To further assess the homogeneity of the SI within the cochlea, an ICC was calculated to assess the agreement between the signal intensities of the basal and apical regions. An ICC value close to 1 indicates high similarity between the apical and basal signal intensities, whereas a value close to 0 means that the values are not similar. The mean SI values of the apical and basal regions of the cochlea contralateral to the tumor were in relative good agreement with each other (ICC, 0.8 [95% confidence interval, 0.75–0.89]).

#### Ipsilateral Cochlea

For the ipsilateral cochleae (tumor side), the mean differences between the apical and basal SI were somewhat larger: 0.6 (SD, 0.7; *p* < 0.001, Wilcox rank sum). This difference is twice as high as the difference on the contralateral side. The ICC was 0.8; however, the 95% confidence interval was relatively broad (0.38–0.93), indicating more variability in SI scores between the basal and apical region on the tumor side. These data might indicate that in some patients, the ipsilateral cochleae have a more inhomogeneous SI pattern compared with the contralateral cochlea.

### Longitudinal Assessment of the Cochlear SI

Over time, the contralateral apical cochlear SI showed a mean difference of −0.1 (SD, 1.8); the basal region showed a mean difference of −0.2 (SD, 2.0). Thus, the mean SI within the cochlear regions shows relatively small differences over time.

For the apical region of the ipsilateral cochlea, the mean difference was −0.1 (SD, 1.1) and for the small or larger basal region, the mean difference was −0.1 (SD, 1.6) and −0.2 (SD, 1.2), respectively. These results are thus comparable to the contralateral side. There were no significant differences in the mean SI scores of the cochleae between the short- and long-term follow-up.

### Ipsilateral Versus Contralateral Cochlear SI

**FIG. 1:**
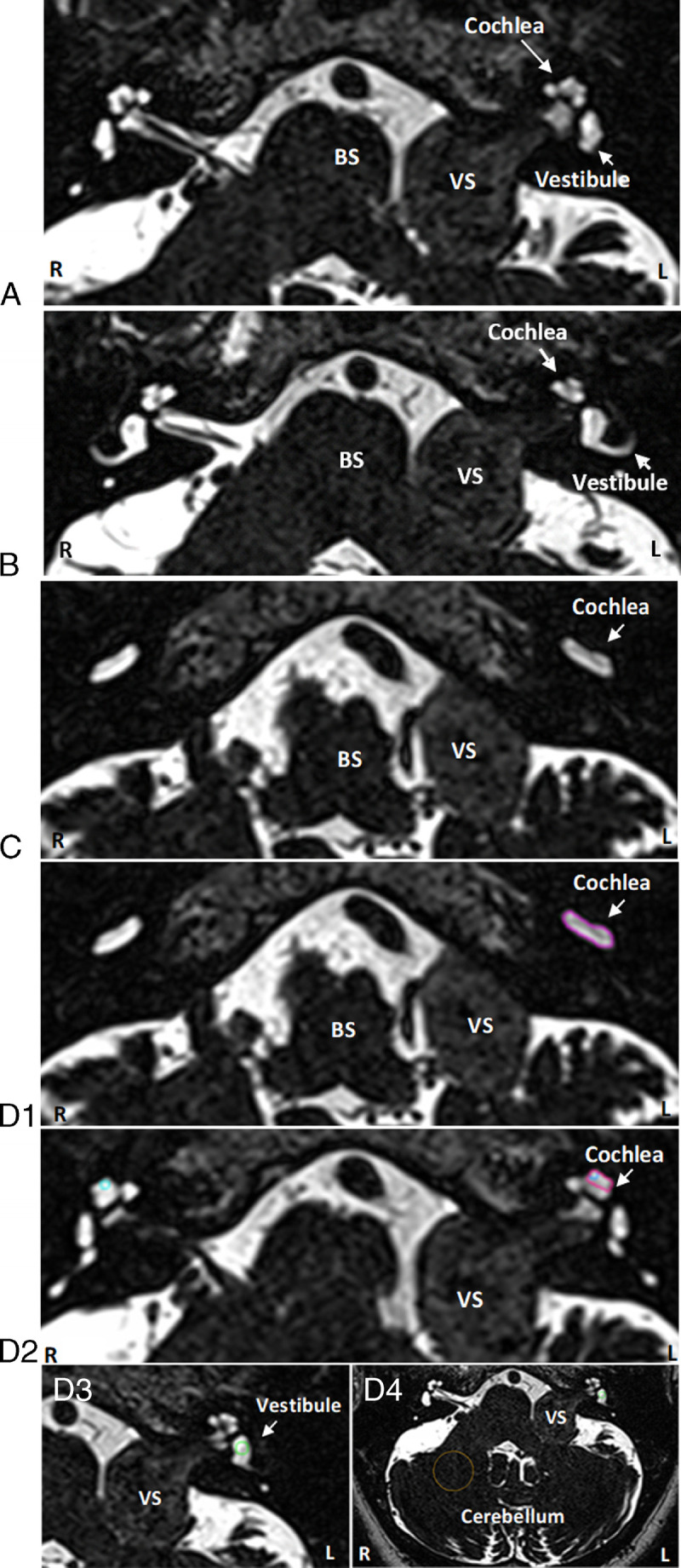
Axial T2-weighted 3D cisternogram MRI at different levels at the cerebellopontine angle from a patient with a left vestibular schwannoma demonstrates decreased signal intensity of the labyrinth on the tumor side. *A*, Decreased signal intensity of the left cochlea compared with the right cochlea, *B* includes a larger part of the vestibules and a semi-circular canal, and *C* basal turns of the cochleae. *D*, 1–4 show delineations of 1) larger basal cochlea region, 2) apical cochlea region, 3) vestibular region, and 4) cerebellar region (delineations in color in the online version). All images are from one patient. BS indicates brain stem; MRI, magnetic resonance imaging; VS, vestibular schwannoma.

**FIG. 2:**
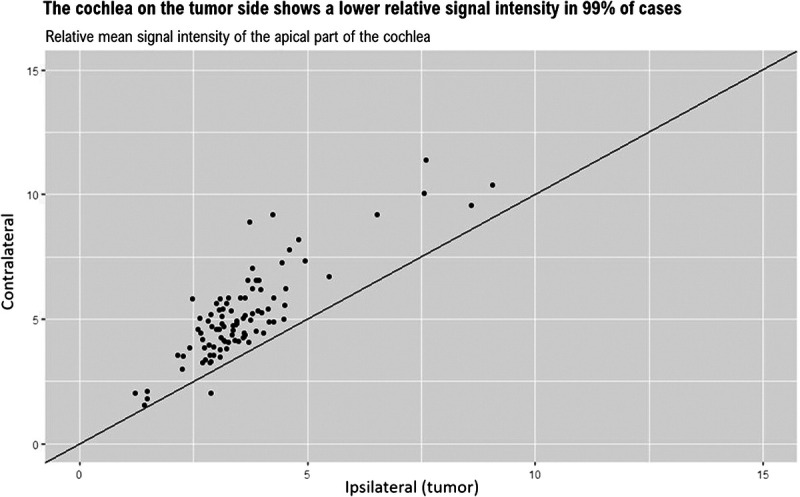
The relative mean signal intensity values of the apical part of the ipsilateral and contralateral cochlea. Linear line has an intercept of zero and a slope of one. The one point showing a higher signal intensity on the ipsilateral side belongs to a patient 15 months after proton radiotherapy who also experienced bilateral vestibular symptoms and some hearing loss on the contralateral side (PTA, 33 dB; SDS, 90%).

Before treatment, 97% to 100% of the ipsilateral cochleae showed a lower SI score compared with the contralateral side (Table 2). An example of an MRI is shown in Figure 1. The percentages remained relatively stable over time, only the small basal cochlear region decreased to 85%, with four or five (dependent of follow-up time point) patients not demonstrating a decreased SI compared with the contralateral side. Figure 2 shows a comparison between the bilateral apical cochleae (all time points included), which demonstrates a lower ipsilateral value in all but one scan. The median percent decrease in SI between the ipsilateral and contralateral cochleae ranged between 21% and 38% at different time points (Table 2). The vestibule showed a smaller median percent decrease at 11% to 16%, and the larger basal cochlear ROI showed a larger median percent decrease between 61% and 67%. The mean difference between the ipsilateral and contralateral cochleae within one MRI scan was 1.6 (SD, 1.0). This difference is much larger than the intracochlear differences as described in the Apical Versus Basal Cochlear SI and Longitudinal Assessment of the Cochlear SI sections and is therefore likely an actual measured difference (and not solely a measurement error).

To further examine a hypothesis that the difference in cochlear SI between the ipsilateral and contralateral sides could (in part) be explained by a more inhomogeneous SI of the ipsilateral side (a selection bias), instead of representing a real difference, a third region of the ipsilateral cochlea comprising a large volume was assessed. This ROI was delineated at the largest basal portion of the cochlea that was visible in one slide. A larger ROI is less dependent on an exact location that an assessor chooses. This region showed an even greater mean difference between the ipsilateral and contralateral cochlea: 2.1 (SD, 1.2; Table 2).

### The Cochlear Versus the Vestibular SI

When compared with the cochlea, the relative mean SI value of the vestibule is higher, regardless of the side (Table 2). This difference, however, is more pronounced on the ipsilateral (tumor) side. The differences between the ipsilateral and contralateral vestibules were much smaller (mean [SD], −0.7 [1.0]) than the cochleae. The vestibule showed a lower SI value on the ipsilateral side in 79%, 69%, and 74% of cases, at pretreatment, and short- and long-term follow-up, respectively.

For the vestibules, the mean difference in relative SI between pretreatment and posttreatment MRI within the same patients was −0.4 (SD, 2.1), which was thus a slightly larger SI difference over time for the vestibules than for the cochleae.

### Hearing Loss and Severe Dizziness

At baseline, the median PTA was 41 dB (IQR, 34–50 dB; range, 3–75 dB), and the median maximum SDS was 78% (IQR, 63–88%; range, 8–100%). After proton radiotherapy, there was an average annual increase in PTA of 11 dB (IQR, 7–18 dB). Progressive SDS hearing loss was seen in 51% of patients at the first follow-up assessment. This percentage increased to 61% at the latest assessment.

**FIG. 3:**
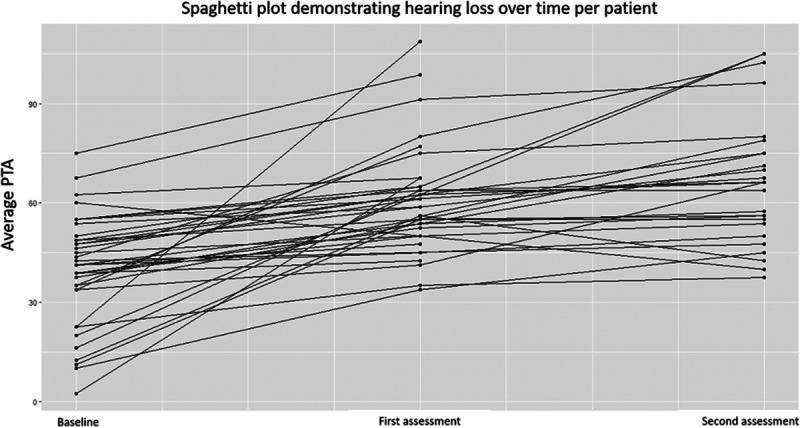
Spaghetti plot demonstrating the change in PTA per patient between baseline and short- and long-term follow-up: Hearing loss increased most prominently between baseline and the first assessment. Follow-up duration until first assessment was a median of 14 months (IQR, 9–17 months); second assessment was 49 months (IQR, 27–62 months). IQR indicates interquartile range; PTA, pure-tone audiometry.

The hearing loss rate was most prominent between the pretherapy (baseline) assessment and the first follow-up assessment thereafter (Fig. 3). The average increase in PTA per year at that point was 15 dB (IQR, 9–21 dB); the regression line in this figure has a *p* value less than 0.001 and an adjusted *R*^2^ of 26% (which explains the amount of variability in the data that is explained by the follow-up time after proton radiotherapy). The hearing loss rate thereafter was much smaller and statistically nonsignificant: on average 2 dB per year (IQR, 0–6 dB) (regression line with a *p* value of 0.2; adjusted *R*^2^ of 1%).

Of the five (of 34) patients who experienced severe dizziness before treatment, one resolved over time and four reported decreased dizziness. In two of these five patients, bilateral vestibulopathy occurred (proven by vestibular testing). One patient without previous dizziness experienced a temporal increase after proton radiotherapy. Because of the low prevalence of new-onset severe dizziness, no statistical analyses were performed.

### The Cochlear SI as a Parameter or Predictor of Hearing Loss

Although the relative cochlear SI remained relatively stable in all patients, average hearing loss increased over time, making a direct or strong correlation between the cochlear SI and hearing loss unlikely. A linear mixed model shows that the impact of the cochlear SI on the PTA was small, with confidence intervals including zero (first model, Table 3). When assessing both ears (while accounting for the clustered nature of the data by adding a random effect for the interaction of side and individual), some effect is seen, but the effect size is deemed insignificantly small.

**TABLE 3:** Outcome of two linear mixed models to test the association between relative apical cochlear signal intensity scores and the pure-tone average

Predictors	Ipsilateral Assessments			Bilateral Assessments		
	PTA in dB			PTA in dB		
	Estimates	95% CI	*p*	Estimates	95% CI	*p*
Intercept	49.73	35.57 to 63.89		44.47	33.86 to 55.08	
Apical cochlear intensity	−0.85	−4.52 to +2.82	0.649	−2.99	−5.19 to −0.79	0.008
Duration of follow-up (in months)	0.27	0.12 to 0.43	<0.001	0.21	0.12 to 0.30	<0.001

The first model (ipsilateral assessment) was corrected for the duration of follow-up, and the second model (bilateral assessment), for the duration of follow-up and side of the measurement. The models show that the impact of the cochlear signal intensity on the PTA was small. The second model, assessing both ears, shows some effect, that is, correlation between cochlear signal intensity and hearing loss, but the effect is small (−2.99 dB per unit increase in cochlear signal intensity).

Furthermore, the relative baseline cochlear SI was found to have no evident predictive value for hearing loss after proton radiotherapy in this cohort (Spearman's *ρ* = 0.23, *p* = 0.12).

## DISCUSSION

In patients with sporadic VS, an evidently lower SI on T2-weighted cisternographic MRI can be seen within the cochlea on the side of the VS, when compared with the contralateral cochlea. The cause of this associated diminished SI is as of yet uncertain. In this study investigating VS patients treated with proton radiotherapy, this diminished cochlear SI is present in almost all patients, already before the start of proton therapy. The SI loss does not increase after proton radiotherapy and remains relatively stable during post-irradiation follow-up, indicating that this phenomenon is associated with the occurrence of a VS rather than being the result of (proton) radiotherapy.

Previous reports have assessed the predictive or cross-sectional relationship between the labyrinthine SI and audiovestibular symptoms in VS patients managed by active surveillance, surgery, or photon radiotherapy. These studies have shown mixed results. For example, one study found a predictive correlation between the hypointense cochlea and hearing loss during follow-up in patients under active surveillance (7). Four other studies reported that a normal cochlear SI may predict better postoperative or postradiotherapy hearing levels (2,4,6,8). The authors suggest that the absence of a decreased cochlear SI may be used as a selection criterion for VS treatments aimed at hearing preservation, but even though the correlations calculated in these reports are statistically significant, the predictive power itself is usually moderate at best. Four other reports failed to find a significant correlation between hearing loss and cochlear SI (3,5,9,16).

These mixed results may be due to publication bias, differences in scoring systems or categorization of end points, and interrater reliability differences. For this study, longitudinal contralateral assessments were used to assess the technique's reliability. We included patients with a wide range of hearing loss severity before and after treatment. However, we found no clear correlation between cochlear SI loss and pretreatment or posttreatment hearing loss. Furthermore, even patients with good hearing (SDS >80%) demonstrated low cochlear SI scores when compared with the contralateral cochlea. The occurrence of diminished cochlear SI on cisternographic T2-weighted imaging also did not seem to be a predictor of future hearing loss after proton radiotherapy. The clinical value of the diminished cochlear SI therefore remains uncertain.

Hearing loss in VS patients is likely caused through a multifactorial process, including compression of the tumor on the vestibulocochlear nerves and labyrinthine artery (which is a terminal artery) and vein (17). However, patients with large VSs do not always have (severe) hearing loss and vice versa, indicating that tumor compression is likely not the only cause of hearing loss (18,19). Another hypothesis for hearing loss in VS patients involves alterations in the composition of the perilymph or endolymph (20,21). Theoretically, protein changes are caused by a breakdown of the blood-endolymph and/or perilymph barrier. This could increase infiltration from plasma due to stasis of blood, which would give protein infiltration into the perilymph (20). Another hypothesis is that the altered protein levels are a result of a cell-mediated immune response to tumor antigens (22,23). If the inner ear fluid's content changes significantly, this could theoretically be made visible on MRI, that is, as a decrease in the cisternographic T2-weighted labyrinthine SI. Interestingly, the ipsilateral vestibule had a higher SI (much closer to the contralateral side) compared with the ipsilateral cochlea, indicating different ipsilateral cochlear and vestibular effects in VS patients. Although an association between a decreased SI of the cochlea or vestibule and vertigo has been reported, we could not reliably assess the correlation between dizziness and the vestibular SI as seen on MRI because of the low prevalence of new-onset vestibular complaints in this cohort (5).

Other suggested MRI biomarkers in VS patients include fluid-attenuated inversion recovery (FLAIR) SI (which is increased on the affected side) and an endolymphatic or utricular hydrops (21,24–28). Results vary, but most studies find small or moderate correlations between the degree of hearing loss and these imaging biomarkers. The current lack of consensus on how to report hearing outcomes and the frequent use of oversimplified outcome measures hamper the comparability of studies looking into imaging biomarkers to predict or explain hearing results (29). Our mixed model included PTA. Arguably SDS is at least as important, as it more closely resembles hearing function in daily life. However, maximum SDSs are less reliable and less sensitive to change than PTA.

This study has some inherent strengths and limitations: It is a retrospective study with a relatively limited sample size because of the rarity of the disease, especially in combination with proton therapy. In addition, patients with hearing or vestibular complaints were more likely to receive audiometry and/or vestibular testing and are therefore probably overrepresented in this study. All of the included patients had an indication for VS treatment (with proton therapy), with the most common indication being tumor progression. This may introduce a selection bias as growing tumors have been reported to be associated with more severe hearing loss (17). Last, MRI scans from different centers, MRI scanners, and MRI manufacturers were included, which is known to hamper comparability. This, however, best reflects clinical practice, and we feel that the methodology proposed in this study (i.e., using relative SI values) is valuable in helping to overcome this limitation. The use of relative SI values furthermore enables (bilateral) longitudinal assessment of signal intensities, which has not been reported before, and a more nuanced evaluation of effect size.

## CONCLUSIONS

The relative mean cochlear SI on high-resolution cisternographic T2-weighted MR imaging in VS patients is diminished on the ipsilateral side, when compared with the ipsilateral vestibule and the contralateral cochlea/vestibule. This suggests specific ipsilateral cochlear effects by the tumor in VS patients. The SI of the ipsilateral cochlea does not further decrease after proton radiotherapy, and a diminished cochlear SI does not correlate with subsequent hearing loss. Thus, the diminished SI seems to be a result of the occurrence of the VS rather than the therapy. Its cause, onset and clinical relevance, however, remain to be elucidated.

## Supplementary Material
