# Supplementary material for: Labyrinthine Fluid Signal Intensity on T2-Weighted MR Imaging in Patients With Vestibular Schwannomas Undergoing Proton Radiotherapy: A Longitudinal Assessment
Source: Otol Neurotol. 2022 Dec 21;44(2):183–90. doi: 10.1097/MAO.0000000000003774 (PMC9835662; doi:10.1097/MAO.0000000000003774)
Supplement: Supplementary file 2 [file on-44-183-s002.docx]

**Supplementary material**

|  | Pre-treatment | Post-treatment I | Post-treatment II |
| --- | --- | --- | --- |
| Median time since proton therapy (IQR) | - | 14 (9-17) | 49 (27 – 62) |
| Magnet Strength in Tesla | 1.16 T: 1 scan  1.5 T: 19 scans  3 T: 14 scans | 1 T: 1 scan  1.5 T: 20 scans  3 T: 13 scans | 1 T: 1 scan  1.16 T: 2 scans  1.5 T: 17 scans  3 T: 7 scans |
| TR (range) | 5.5 – 7381 | 4.7 – 5862 | 5.4 – 6262 |
| TE (range) | 2.2 – 2608 | 2.2 – 175045 | 2.2- 172703 |
| Matrix | 1: 512 (256 - 960)  2: 512 (256 - 960)  3: 64 (30 – 156) | 1: 512 (320 - 960)  2: 512 (320 - 960)  3: 72 (40 – 124) | 1: 512 (256 - 960)  2: 512 (256 - 960)  3: 74 (36 – 132) |
| Slice thickness | 0.5 (0.15 – 1.0) | 0.5 (0.4-1.5) | 0.5 (0.2-1.0) |

**Supplementary Table 1** MRI characteristics

Abbreviations: IQR = inter quartile range, TR = repetition time, TE = time to echo.

|  | **Pre-treatment** | **Short-term follow-up** | **Long-term follow-up ^a^** |  |
| --- | --- | --- | --- | --- |
|  | *Median (IQR)* | *Median (IQR)* | *Median (IQR)* | |
| **Ipsilateral** |  |  |  | |
| Cochlea apex ROI | 6685 (2234 – 46489) | 3072 (481 – 13010) | 1248 (466 – 9930) | |
| Cochlea basal ROI | 5716 (1829 – 38332) | 2697 (377 – 9363) | 806 (400 – 8066) | |
| Vestibular ROI | 6498 (2230 – 40711) | 3755 (541 – 11752) | 1254 (466 – 9262) | |
| Cochlea larger basal ROI ^b^ | 4474 (1650 – 34132) | 3020 (288 – 6970) | 757 (318 – 6697) | |
| **Contralateral** |  |  |  | |
| Cochlea apex ROI | 6685 (2234 – 46489) | 3072 (481 – 13010) | 1248 (466 – 9930) | |
| Cochlea basal ROI | 7163 (2652 – 52731) | 3649 (510 – 11417) | 1270 (300 – 9853) | |
| Vestibular ROI | 8265 (2683 – 50212) | 4161 (665 – 12815) | 1444 (516 – 9513) | |
| **Cerebellum** |  |  |  | |
|  | 1642 (625 – 11214) | 855 (107 – 2364) | 497 (70 – 1769) | |

**Supplementary Table 2** absolute region of interest (ROI) values

^a^ Twenty-seven out of 34 patients were available for the third assessment.

b As the cochlear signal intensity ipsilateral to the tumor was less homogeneous (paragraph 3.2.2) than that of the contralateral cochlea, a second and larger part of the ipsilateral basal cochlea was delineated because in doing so, the measured signal intensities were less dependent on the specific place of delineation within the cochlea.

Abbreviations: ROI = region of interest, SD = standard deviation
